# AI am a rheumatologist: a practical primer to large language models for rheumatologists

**DOI:** 10.1093/rheumatology/kead291

**Published:** 2023-06-12

**Authors:** Vincenzo Venerito, Emre Bilgin, Florenzo Iannone, Sedat Kiraz

**Affiliations:** Rheumatology Unit, Department of Precision and Regenerative Medicine—Ionian Area, University of Bari ‘Aldo Moro’, Bari, Italy; Division of Rheumatology, Department of Internal Medicine, Hacettepe University Faculty of Medicine, Ankara, Turkey; Rheumatology Unit, Department of Precision and Regenerative Medicine—Ionian Area, University of Bari ‘Aldo Moro’, Bari, Italy; Division of Rheumatology, Department of Internal Medicine, Hacettepe University Faculty of Medicine, Ankara, Turkey

**Keywords:** Natural language processing (NLP), large language models (LLMs), generative pre-trained transformers (GPT), academia, rheumatology, opportunities, challenges

## Abstract

Natural language processing (NLP), a subclass of artificial intelligence, large language models (LLMs), and its latest applications, such as Generative Pre-trained Transformers (GPT), ChatGPT, or LLAMA, have recently become one of the most discussed topics. Up to now, artificial intelligence and NLP ultimately impacted several areas, such as finance, economics and diagnostic/scoring systems in healthcare. Another area that artificial intelligence has affected and will continue to affect increasingly is academic life. This narrative review will define NLP, LLMs and their applications, discuss the opportunities and challenges that components of academic society will experience in rheumatology, and discuss the impact of NLP and LLMs in rheumatology healthcare.

Rheumatology key messages

- Artificial intelligence (AI) is expanding its role in medicine, including rheumatology.
- Natural language processing and large language models offer possibilities and challenges in academic settings.
- Responsible use of AI systems is crucial, considering limitations, safety issues and ethical concerns.

## Introduction

For centuries, as humans, we kept learning the basics of life and used all the data to design our future, sometimes in a positive way, other times in a catastrophic way. Nevertheless, in the last century, we ‘invented’ a game-changer (computers) that seems to have unlimited potential even to change our thinking. The idea of artificial intelligence (AI) came up hand in hand with the invention of computers. As hardware and software technologies are improving day by day and every segment of society appreciates the value of ‘data’, AI is enlarging its place in our lives, and so it is in medicine. AI has been used in medicine for several decades: image/case identification, classification, scoring and grading, education, analysing large data, etc. [1, 2]. With recent developments, AI is now ‘invading’ our minds in another way: natural language processing (NLP) [3, 4]. Nowadays, one of the most attractive topics is an application of NLP: Generative Pre-trained Transformer (Chat-GPT) [5]. In this editorial, we will explore, and sometimes speculate upon, the chances and risks emerging using Chat-GPT and other similar tools in rheumatology in academic and clinical settings.

## What is natural language processing?

Artificial intelligence can be defined as the ability of computers and related systems to execute tasks that mainly require the human mind and intelligence, such as learning, reasoning, decision-making, understanding, recognizing, and natural language processing (NLP). Machine learning, expert systems, speech recognition, planning, robotics, vision, and NLP processing can be counted as the subsets of AI [2]. NLP can be considered as the bridge of communication between machines and humans. With the improvements in deep learning algorithms and neural networks, the abilities of NLP will exponentially progress. Key components of NLP are *text analytics* (extracting meaningful information from unstructured text data), *speech recognition* (recognizing and transcribing spoken language), *natural language understanding* (understanding and interpreting human language in a particular context), *natural language generation* (generating human-like language) and *machine translation* (translating text from one language to another). Generative Pre-trained Transformer (GPT), its chatbot (Chat-GPT) and Meta’s Large Language Model Meta AI (LLaMA) are applications of NLP and are the hot topics of the current times [5, 6].

## What are Generative Pre-trained Transformers, its chatbot and LLaMA

Large language models (LLMs) are deep learning models trained on huge amounts of text data to generate human-like text based on context and input. Technically, LLMs use advanced NLP techniques, such as tokenization, parsing and named entity recognition (NER) [5]. Tokenization breaks the text into individual words or tokens while parsing analyses the grammatical structure to identify relationships between the tokens. NER locates and classifies named entities, which might also refer to medical conditions and medications. The extracted data can then be structured, aggregated and analysed for actionable insights. GPT are large language models constructed via deep learning methods (namely transformers) first introduced in 2018 by the OpenAI company. The latest version (GPT-4) was recently released in the middle of March 2023. The model has been pre-trained in an enormous amount of unlabelled text data via unsupervised deep-learning techniques and then fine-tuned with reinforced supervised learning from human feedback. Training data consisted of online books, published articles, medical case reports and case series, web pages, social media and online forums up to the end of 2021. GPTs are considered a part of generalized AI, meaning they are not trained for specific tasks. The output is generated in a probabilistic manner in that the model selects the most likely one based on the internal dynamics of the model and input. Interestingly, the models are constantly learning from the inputs and outputs.

ChatGPT is the interactive, conversational interface of the GPT and was released in November 2022 by OpenAI for public use [5]. For now, it is based on GPT-3.5 in the free version and GPT-4 in the paid version. It mainly works with the same principles as GPT: processes inputs, generates multiple possible responses, selects the most likely one, and presents it. Also, ChatGPT remembers what was said earlier in the conversation and replies according to the context of the conversation in a continuous manner. By ChatGPT, we can communicate with machines in a human-like manner and use the power of AI more efficiently.

LLMs of medical interest are not limited to GPT-4. Meta recently released Large Language Model Meta AI (LLaMA), aiming to be more efficient and less resource-intensive compared with other models, increasing its use for a broader base [6]. LLaMA is particularly notable for its availability under a non-commercial licence for researchers and organizations, facilitating its use in various projects. Like GPT, LLaMA is based on a transformer architecture to analyse massive data volumes and generate new content or make predictions based on the data. Another noteworthy difference is their training data. LLaMA is trained on a diverse text corpus, including scientific articles and news articles, while GPT-3.5 is primarily trained on internet text, such as web pages and social media content. As a result, LLaMA may be better suited for generating technical or specialized language, whereas GPT-3.5 may excel in producing informal or conversational language. LLaMA is available in a variety of sizes, ranging from 7 billion to 65 billion parameters (B). The larger models are more powerful, but they also require more computational resources to train and use. LLaMA can run in a local machine and be fine-tuned to improve its performance on specific tasks; this can be done by providing the model with additional data and feedback (Table 1).

**Table 1.** Comparison of general features of GPT-4 and LLaMA

Feature	LlaMA [6]	**GPT-4 [** 5 **]**
**Origin**	Developed by Meta	Developed by OpenAI
**Model size**	Four versions available according to billion parameters (B): 7B, 13B, 33B, 65B.	>1 trillion parameters (expected)
**Computational demands**	Models can be run and fine-tuned on local machines	Dedicated systems based on several high-performance Nvidia A100 Graphics Processing Units
**Training data**	Diverse range (e.g. scientific articles, news); it can be easily fine-tuned for accomplishing specific task.	Primarily internet-based text (e.g. web pages)
**Language specialization**	More technical or specialized language	More informal or conversational language
**Accessibility**	Non-commercial license	Licensing terms and conditions from OpenAI

## GPT and ChatGPT in academic life in rheumatology: challenges and possibilities

How can AI aid rheumatologists in their activity? Applications of generative AI will certainly affect almost all sectors. We can just see the tip of the iceberg for now, but we will soon understand its application in the near future. Before diving into the possible applications of GPT and ChatGPT in academic life, we would like to underline the precautions to be always kept in mind.

- All components of the academic community (e.g. authors, editors, publishers, researchers, trainees) should be aware of the basics and the developments in AI.
- Whatever was generated using generative AI, humans should always proofread and supervise the content generated.
- Users of these systems should always keep in mind the limitations and challenges of these systems and use the systems responsibly.

## Limitations and challenges

The current version of GPT has many limitations and challenges (especially regarding safety issues). With the improvements in technology, limitations will decrease; however, safety issues are always in front of us [6]. Below we will try to list some of the limitations and challenges regarding academic life:

- The system tends to produce incorrect, unreal contents (hallucination). From an academic perspective, the system may create non-existant citations, which is very dangerous. In addition, as the system improves, outputs tend to be more convincing and over-relying on themselves. Also, the competition between AI companies may lead to less control of the content and outputs.
- The system can provide harmful content, which may cause violent and discriminatory language, although several precautions have already been taken.
- Because of the large and very diverse content for educating the system, the system can create biased content.
- The personal data of individuals can be uncovered; also, private data regarding the publication process of an article may be altered.
- Trained data is up to 2021 (for now), so recent advancements in the literature are missing in the model.
- Ethical issues: authorship, plagiarism, and so on. In the current definition of ICJME, we think that any AI system cannot be considered an author. So, it needs to be defined how we will place and nominate the system: associate author? author assistant? Besides, a few publishers have declared that they will not accept ChatGPT as an author, and they recommended that authors using ChatGPT declare it in the methods or acknowledgments. Plagiarism is also another issue. The system may create similar outputs with similar queries. So, boundaries of plagiarism in the era of AI need to be re-shaped.

## Opportunities

The opportunities that AI offers are already amazing [7]. From an academic perspective, AI suggests solutions from generating research questions to advertising published articles and so on. The OpenAI Application Programming Interface (API) allows the deployment of advanced models like GPT-3.5, or even GPT-4 (given limited access permission), to create task-specific applications tailored for academic purposes. Mainly, it saves time and allows researchers to focus on their work's content. Here, we tried to list some of the possibilities and opportunities:

- It can suggest research questions and hypotheses related to specific or general topics. However, the system is not trained with recent data, so that outputs may have already been studied. It can contribute to all sections of an article.
- It can help with methodological problems. It can suggest study designs and sizes and define dependent and independent variables. It can suggest designs for follow-up studies. For statistical analyses, it can suggest relevant statistical methods regarding how to test hypotheses, write codes for statistical packages like R and/or python, and correct mistakes in the code.
- The system can create and suggest abstract and title alternatives.
- It can assist in translation, editing, proofreading, correcting grammatical errors, and improving the readability and overall quality of the manuscript.
- As a supervising agent, one AI model can check whether a given content includes outputs from ChatGPT. DetectGPT can effectively assess whether the content was written by human or ChatGPT.
- In the peer review process, the system can critically appraise the manuscript and assess the methodology, validity and relevance of the citations. Also, it can provide insights into possible conflicts of interest, plagiarism and other ethical problems. An OpenAI API-based package called ‘PaperQA’ (https://github.com/whitead/paper-qa) is used for questioning and getting answers from texts and can be used to review papers effectively.
- After publication, it can create advertisement texts and figures. It can also create formal e-mails, social media posts, and educational materials.
- The system can assist in writing ethical committees and grant applications.
- The above-mentioned opportunities and challenges converge on the same point: AI systems should always be used responsibly, and humans should supervise all steps.

## Healthcare applications

Large language models like GPT-4 or LLaMA have significant potential in rheumatology, offering a range of applications and benefits. They can assist in processing and analysing vast amounts of medical literature, letting clinicians and researchers to stay up-to-date with the latest advancements in rheumatology. Additionally, these models can help create personalized patient education materials, ensuring that patients receive accurate and easy-to-understand information about their conditions and treatments. They can also be used for automating routine tasks such as documentation, by analysing patient data, treatment details and follow-up recommendations, LLMs create well-structured, coherent and accurate discharge letters, allowing healthcare professionals to focus more on patient care. Moreover, with proper finetuning and adequate validation, they can potentially contribute to the development of decision-support tools, aiding rheumatologists in diagnosing and managing complex rheumatic diseases more effectively.

LLMs, like GPT-4 or LLaMA may also reduce time-to-referral. In fact, they can potentially be used to analyse text data from forums, social media or electronic health records to identify patterns or keywords associated with rheumatic diseases. By leveraging natural language processing capabilities, LLMs can help recognize early signs or symptoms of rheumatic conditions and facilitate early referral to specialists. In this regard, one potential use is the extraction and analysis of data from general practitioner (GP) healthcare records (Table 2). In this context, LLMs can be employed to analyse unstructured text data and extract valuable insights to improve patient care, detect patterns and even identify early signs of diseases. However, privacy and data security concerns need to be addressed, as handling sensitive personal information in this context requires strict adherence to data protection regulations and ethical guidelines.

**Table 2.** GPT-4 *vs* LLaMA 7B *vs* LLaMA 13B: comparing rheumatology referral recommendations scraped from hypothetical general practitioner records

Patient 1			
To which specialist should I refer this patient?			
Female, 24 years old			
From adolescence: malar rash			
2015: leucopenia and low C3			
Tenderness of both wrists today			

**GPT-4**		**LLaMA 7B**	**LLaMA 13B**

Considering the patient's history of malar rash, leucopenia, low C3 levels, and current wrist tenderness, it is recommended to refer this patient to a rheumatologist. These symptoms and laboratory findings could suggest an autoimmune condition such as systemic lupus erythematosus (SLE) or another connective tissue disease. A rheumatologist has the specialized knowledge and experience to evaluate, diagnose, and manage such conditions.		The patient should be referred to rheumatologist.	The patient should be referred to rheumatologist for futher investigation and treatment.

**Patient 2**			

To which specialist should I refer this patient?			
Male, 24 years old			
From adolescence: recurring fever and diarrhea lasting 8–9 days			
2017: pericarditis			

**GPT-4**	**LLaMA 7B**		**LLaMA 13B**

GPT-4: Given the recurring fever, gastrointestinal symptoms, arthralgia and history of pericarditis, this patient may need to be evaluated by a few specialists: Rheumatologist: The pattern of recurring fever and pericarditis may point towards an autoinflammatory or autoimmune condition, which is within the purview of a rheumatologist. Gastroenterologist: Persistent diarrhea warrants an evaluation by a gastroenterologist, especially if it is a chronic issue. They can perform diagnostic procedures like a colonoscopy or endoscopy to look for causes. Cardiologist: The history of pericarditis may also require ongoing management or follow-up with a cardiologist. Infectious Disease Specialist: If the cause of the recurring fevers is not clear, an infectious disease specialist could be helpful in ruling out chronic or recurrent infections.	This patient should be referred to a specialist in infectious diseases.		I would refer this patient to a rheumatologist, and I would also refer this patient to a gastroenterologist.

GPT-4 appears to offer a more comprehensive and detailed analysis of the presented patient symptoms, suggesting both potential diagnoses and appropriate specialist referrals. On the contrary, LLaMA 7B provides more succinct responses, aiming to pinpoint suitable specialist referrals without an elaborate reasoning process. Although both models seem practically equivalent in identifying common conditions like systemic lupus erythematosus, LLaMA 7B falls short in recognizing less common conditions such as autoinflammatory diseases. The utilization of a more complex model (13B), enhances performance, even in absence of specific fine-tuning. GPT-4 accessed on 29 May 2023.

LLaMA 7B: A Local LLaMA 7B run locally on an Apple M1Max 64GB RAM, fine-tuned on Stanford Alpaca database using low-rank adaptation. Hyperparameter tuning: temperature 0.22, top P 0.25, top K 40.

LLaMA 13B: A Local LLaMA 13B run locally on an Apple M1Max 64GB RAM, Hyperparameter tuning: temperature 0.22, top P 0.25, top K 40.

While working with sensitive patient data, it is crucial to address potential privacy and data security concerns. Compliance with data protection regulations, such as the Health Insurance Portability and Accountability Act (HIPAA) in the US or the General Data Protection Regulation (GDPR) in the European Union, is essential to ensure the ethical use of LLMs for data scraping in healthcare. Furthermore, data anonymization techniques, like removing personally identifiable information (PII) and employing differential privacy methods, can help protect patient privacy while still allowing for meaningful data analysis. A locally hosted, fine-tuned LLM like LLaMA could potentially address some of the ethical concerns related to privacy and data security in healthcare settings. By deploying the LLM within a hospital’s secure infrastructure, data would be processed internally, reducing the risk of unauthorized access or data breaches compared with cloud-based solutions. Finetuning the LLM on hospital-specific data would allow it to better understand the unique context and terminology used within that institution. It could also be tailored to comply with the hospital's data handling policies and relevant data protection regulations.

However, it is important to note that implementing a local LLM does not completely eliminate all ethical concerns. Ensuring the responsible use of the LLM still requires adherence to data protection regulations, such as HIPAA or GDPR, and the application of data anonymization techniques when necessary. Furthermore, the development and maintenance of the LLM should follow guidelines for responsible AI, including transparency, fairness and accountability.

The potential biases in medical data used to train LLMs can negatively impact patient care if the models perpetuate these biases in their outputs. Ensuring fairness and addressing biases are vital for maintaining care quality and avoiding harm to patients. Additionally, obtaining informed consent from patients whose data is used for LLM training is essential, as they must be aware of the intended use of their data and potential risks associated with its use in LLMs.

In light of the growing integration of LLMs in healthcare, it is vital to establish clear policies and agreements between healthcare providers, researchers, technology developers and patients. Ensuring responsible and ethical patient data usage, as well as maintaining transparency and accountability, is critical for addressing potential negative consequences in a timely and responsible manner.

On 30 March 2023, the Italian Data Protection Authority (Garante) issued a provisional measure against OpenAI, asking to temporarily suspend ChatGPT’s processing of personal data for individuals in Italy and ultimately leading OpenAI to disable service for Italian users. This unprecedented action was taken ahead of the Garante’s investigation into ChatGPT’s privacy practices, following a data breach that exposed users’ information. The Garante identified potential GDPR violations, including a lack of transparency in data processing, inaccuracies in data processing, and failure to verify users’ ages. Even if the ban was removed on 28 April 2023, the case has gained significant attention due to ChatGPT’s rapid growth and marked the first instance of an EU data protection authority intervening in the data processing activities of a widely used generative AI tool [8]. Although the concerns raised by the Garante seem legitimate, international AI regulation and a shared approach should be preferable to avoid draconian decisions.

Ultimately, there is an urgent need for patients and rheumatologists to understand the potential and limitations of AI in healthcare. Both must have a clear understanding of how these technologies work. This knowledge fosters trust and acceptance of AI, improving patient outcomes and overall healthcare efficiency. AI is not intended to replace healthcare professionals but to augment their abilities. Understanding AI allows doctors and patients to collaborate better with the technology, maximizing its potential while maintaining a human-centric approach to care.

## Data Availability

No new data were generated or analysed in support of this article.
